# The Role of MicroRNAs in Treg‐Derived Extracellular Vesicles: A Narrative Review Focusing on Immune‐Related Diseases

**DOI:** 10.1155/jimr/6798693

**Published:** 2026-03-26

**Authors:** Yudong Gao, Juncheng Zhou, Yuwen Mao, Mingxuan Yuan, Reyihanguli Yimin, Fengbo Zhang

**Affiliations:** ^1^ The Second Clinical Medical College of Xinjiang Medical University, Urumqi, 830011, Xinjiang, China; ^2^ State Key Laboratory of Pathogenesis, Prevention and Treatment of High Incidence Diseases in Central Asia, The First Affiliated Hospital of Xinjiang Medical University, Urumqi, China, xjmu.edu.cn

**Keywords:** extracellular vesicles, immune regulation, immune-related diseases, microRNAs, regulatory T cells

## Abstract

Regulatory T cells (Tregs) are a unique class of immunosuppressive cells that play a key role in managing immune‐related diseases. Growing evidence indicates that the immunosuppressive effects of Tregs also rely on cell‐to‐cell interactions that are facilitated by the secretion of extracellular vesicles (EVs). Treg‐derived EVs (Treg‐EVs) are lipid bilayer vesicles that are released from Tregs and have been shown to promote intercellular communication and facilitate immune regulation by transferring various messenger RNAs (mRNAs), microRNAs (miRNAs), and proteins. miRNAs are small, noncoding RNA molecules that regulate gene expression after transcription and can influence multiple target genes by binding to the 3′‐untranslated region (UTR) of target mRNAs to exert influence on a wide array of cellular functions. Although the roles of miRNAs and Treg‐EVs in various diseases have been extensively studied, the regulatory functions of the different miRNAs within Treg‐EVs in immune‐related diseases remain largely unexplored. In this narrative review, we investigate the functional roles of Tregs and their derived EVs in immune regulation. Then, we discuss the specific roles of miRNAs within Treg‐EVs in the context of rheumatoid arthritis (RA), inflammatory bowel disease (IBD), autoimmune vasculitis, multiple sclerosis (MS), and kidney and skin allografts by investigating recent research in autoimmune diseases and transplantation. In these situations, different Treg‐EVs can support the treatment of immune‐related conditions by delivering miRNAs to specific immune cells and promoting immunosuppression through multiple mechanisms.

## 1. Introduction

Regulatory T cells (Tregs) form a specific subgroup of T lymphocytes that exhibit immunosuppressive abilities. These cells play a crucial role in maintaining self‐tolerance and controlling overactive immune responses [1]. As research on extracellular vesicles (EVs) advances, attention has shifted to EVs that are secreted by Tregs; these EVs have become a key focus area within the broader field of immune regulation [2–4]. EVs are nanoscale particles encapsulated in lipid bilayers released by cells and carry bioactive molecules such as proteins, nucleic acids (e.g., mRNA and microRNAs [miRNAs]), and metabolites, serving as key mediators of information transfer between cells [5]. Therefore, EVs are considered as promising tools for the diagnosis and treatment of disease due to their improved stability, targeted properties, and diminished immunogenic responses [6].

miRNAs are a type of noncoding RNA of approximately 22 nucleotides in length that regulate gene expression by binding to the 3′‐untranslated region (UTR) of target messenger RNAs (mRNAs) to participate in cell proliferation, differentiation, and apoptosis [7, 8]. Specific miRNAs in Treg‐derived EVs (Treg‐EVs) can target immune cells such as effector T cells (Teffs) and dendritic cells (DCs) to mediate immunosuppression. For example, the presence of let‐7d in Treg‐EVs has been demonstrated to reduce the inflammatory response by suppressing COX‐2 expression in Th1 cells. Furthermore, it has been observed that miR‐150‐5p and miR‐142‐3p promote the tolerant phenotype in DCs, enhance IL‐10 secretion, and inhibit IL‐6 production [9, 10], while miR‐709 and miR‐2861 have been shown to promote motor recovery after spinal cord injury (SCI) by inhibiting microglial pyroptosis and repairing the blood–spinal cord barrier (BSCB) [11, 12]. Collectively, these findings highlight the unique role of Treg‐EVs and their miRNAs in immune regulation, thus providing a foundation for their application in autoimmune diseases and transplant rejection.

While Tregs, EVs, and miRNAs have been extensively studied, the regulatory functions of distinct miRNAs in Treg‐EVs across immune‐related diseases remain have yet to be elucidated; existing research lacks a unified mechanism‐centric framework connecting miRNA targeting, downstream signaling, and therapeutic potential [13–16]. To address this gap in knowledge, this narrative review first summarizes the core immunomodulatory roles of Tregs and Treg‐EVs and then dissects the context‐specific functions of Treg‐EVs miRNAs in rheumatoid arthritis (RA), inflammatory bowel disease (IBD), autoimmune vasculitis, multiple sclerosis (MS), and allografts, emphasizing their shared and disease‐specific molecular mechanisms, with a focus on their therapeutic potential in autoimmune diseases and transplant rejection, thus providing a reference for generating new insights in this area.

## 2. Tregs and Treg‐EVs

### 2.1. Functional Roles of Tregs in Immune Regulation

**Figure 1:**
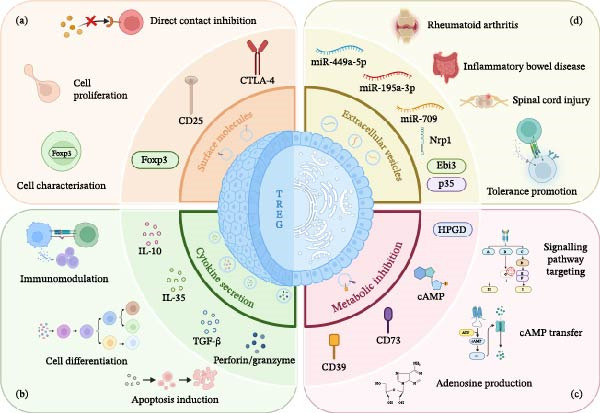
Four major regulatory mechanisms of Treg cells in immune suppression. (a) Surface molecules, such as CD25, CTLA‐4, and intracellular transcription factors, such as Foxp3, which are crucial for cellular characteristics, proliferation, and direct contact inhibition, respectively. (b) Cytokines, such as IL‐10, TGF‐β, IL‐35, and perforin/granzyme, secreted by Treg cells, are involved in immunosuppression, cell differentiation, and the induction of apoptosis in target cells. (c) Metabolites related to Treg cells, including CD39, CD73, cAMP, and HPGD, which influence immune regulation by modulating cellular metabolism and signaling pathways. (d) A variety of microRNAs in Treg‐derived extracellular vesicles may also support the treatment of various immune‐related diseases. cAMP, cyclic adenosine monophosphate; HPGD, hydroxyprostaglandin dehydrogenase.

Tregs are categorized by their origin into natural (nTregs) and inducible (iTregs) subsets. Thymus‐derived nTregs constitutively express CD4, CD25, and Foxp3 [17], restraining the overactivation and proliferation of Th/CTL cells and the functionality of B cells [18]. Conversely, iTregs (including Tr1, Th3, and iTr35 subsets) mediate immunoregulation by secreting IL‐10, TGF‐β, and IL‐35, respectively [17]. Tregs dysregulation, manifested as reduced numbers or functional defects, is known to closely underlie the pathogenesis and progression of immune‐related disorders [19–21], with their immunosuppressive activity orchestrated by cell–cell contact, the secretion of anti‐inflammatory cytokines, and metabolic interference [22, 23] (Figure 1).

Tregs exert suppressive effects via contact‐dependent pathways (CTLA‐4, PD‐1/PD‐L1, and CD25) [17, 24, 25] and cytokines (IL‐10, TGF‐β, and IL‐35) [26–30], and by modulating extracellular adenosine (CD39/CD73) and cAMP [31–33]. Notably, human Tregs express high levels of HPGD, the expression of which is driven by the IL‐2‐activated JAK3‐STAT5 pathway and direct Foxp3 binding, which catalyzes the conversion of prostaglandin E2 (PGE2) to 15‐keto‐PGE2, ultimately inhibiting Teffs via the activation of peroxisome proliferator‐activated receptor‐γ (PPARγ) [34]. In addition, Tregs are also known to exert regulatory effects via direct/indirect pathways and the secretion of EVs (Figure 1).

### 2.2. EVs and the Functional Roles of Treg‐EVs in Immune Regulation

Extracellular particles (EPs) are heterogeneous populations of structures released by cells into the extracellular space; membrane‐bound EVs represent a key subset of EPs [35, 36]. According to the Minimal Information for Studies of EVs 2023 (MISEV2023) guidelines, as summarized in Table 1, EPs are formally classified into two major categories: EVs (membrane‐enclosed structures) and non‐vesicular EPs (NVEPs; nonmembranous particles) [13]. Of these, EVs are primarily categorized based on their physical properties (predominantly diameter) into small EVs (sEVs, <200 nm) and large EVs (lEVs, >200 nm). This diameter‐based classification is recommended as the primary standard by the MISEV2023 guidelines to address the lack of absolute specific markers for EV subtypes, thus avoiding ambiguity in terminology and classification [13].

**Table 1:** Classification and characteristics of extracellular vesicles.

Term	Definition	Category	Diameter (nm)	Usage
EPs				
EVs	Particles that are released from cells, are delimited by a lipid bilayer, and cannot replicate on their own	sEVs	<200	Recommended, but caution required
		Exosome	A subtype of small EVs, usually <200	Discouraged unless subcellular origin can be demonstrated
		lEVs	>200	Recommended, but caution required
NVEPs	Multimolecular assemblies that are released from cells and do not have a lipid bilayer (non‐vesicular extracellular particle fraction)	Proteins, nucleic acids, lipids (if present, do not form a delimiting bilayer membrane)		Recommended

*Note:* There are some terms that have not yet been fully characterized. The measured diameter depends on the specific characterization method. There are no strict size or marker limits between the different classifications, and diameter is not the only standard used to categorize extracellular vesicles.

Abbreviations: EPs, extracellular particles; EVs, extracellular vesicles; lEVs, large EVs; NVEPs, non‐vesicular extracellular particles; sEVs, small EVs.

Although the MISEV2023 guidelines provides timely and appropriate classification and nomenclature for EVs, many of the studies included in this review pre‐date these recommendations and, therefore, do not strictly adhere to these guidelines. To ensure accurate citation and analysis of experimental findings, this review follows the original descriptions of EVs as presented in the primary literature. However, we have also analyzed and categorized all relevant EV data according to the latest MISEV2023 standards, as summarized in Table 2. Notably, the MISEV2023 guidelines recommend the preferential classification of EVs based on biogenesis and diameter, rather than by protein markers, as no single protein is unique to a specific EV subtype [13, 41, 42]. Furthermore, minimal characterization of EVs, including particle/protein quantification, morphological analysis, and the detection of protein markers, along with the use of negative markers (e.g., calnexin and GM130) is mandatory for the valid assessment of EVs to exclude contamination by intracellular organelles [43, 44].

**Table 2:** The application of Treg‐EVs derived microRNAs in immune‐related diseases.

Species/source of Tregs	Isolation method	Validation	Original terms of EVs in the literature	Size of EVs	Terms of EVs (MISEV2023)	MicroRNA	Diseases	Recipient cell	Target	Endpoint and readout	Treg‐EVs‐miRNAs function	References
Mice, spleen, lymph nodes, or tissue	dUC + kit (ExoQuick solution)	DLS, FCM and ELISA (CD63, CD9, CD81)	Treg‐Exos	20–100 nm	EVs	Let‐7d	Colitis and systemic inflammation	Th1 cells	Cox‐2 (Ptgs2)	Th1 cell function + colitis severity: Th1 proliferation (CellTrace Violet), IFN‐γ (ELISA), colon histology (AB‐PAS staining), body weight monitoring	Inhibition of Th1 cell proliferation and IFN‐γ secretion	Okoye et al. [9]
Rat, lymph nodes	dUC	TEM, western blot (CD63, TSG101)	dnIKK2‐Treg‐EV	40–100 nm	sEVs	miR‐503	Kidney allograft	T cells	CCNE1, CCND1	T cell proliferation/cell cycle + allograft survival: T cell proliferation (^3^H‐thymidine/MLR), cyclin E/D1 (western blot), serum creatinine, graft survival (Kaplan–Meier)	Anti‐proliferation by downregulating T‐cell cyclin E and D1 proteins and prolong allograft survival	Aiello et al. [37]
B6 mice	dUC + kit (ExoQuick‐TC solution)	TEM, NTA	Treg‐EVs	Around 100 nm	sEVs	miR‐150‐5p, miR‐142‐3p	N/A	DCs	c‐Myb, IL‐6 mRNAs	DC phenotype + cytokine profile + miRNA transfer: CD80 (FCM), IL‐6/IL‐10/TNF (CBA/ELISA), miR‐150/142 transfer (qPCR)	Increase IL‐10 and decrease IL‐6 following LPS activation, and induce tolerogenic DCs	Tung et al. [10]
Human	dUC + kit (ExoQuick‐TC solution)	TEM, NTA, western blot (CD63, CD81)	Treg‐EVs	mean size of 150 nm, mode of 125 nm	sEVs	miR‐369‐3p, miR‐376c‐3p, miR‐195‐3p	Skin allograft	Teffs	IFN‐γ, IL‐2, IL‐6 mRNAs	Teffs function + graft histology: Teffs proliferation (CellTrace Violet), IFN‐γ/IL‐2/IL‐6 (CBA/ELISA), graft histopathology (H&E), immune cell infiltration (IF)	Modulate Teffs cytokine production by targeting the 3′UTR of IFN‐γ, IL‐2, and IL‐6 mRNAs	Tung et al. [38]
BALB/c mice, spleen	dUC	TEM, NTA, western blot (CD63, TSG101)	Treg‐Exos	30–200 nm	sEVs	miR‐195a‐3p	IBD	YAMC cells	Caspase 12	Colonic epithelial proliferation/apoptosis + IBD inflammation: YAMC proliferation (MTT)/apoptosis (Annexin V/PI), TNF‐α/IL‐1β/IL‐6 (ELISA), colon length, ZO‐1 (IHC)	Targeting pro‐apoptotic Caspase 12 promotes proliferation and inhibits apoptosis in colonic epithelial YAMC cells	Liao et al. [39]
C57BL/6J mice, spleen	EVs isolation kits	TEM, NTA, western blot (CD9, CD63, CD81, TSG101)	iTreg‐EVs	40–160 nm	sEVs	miR‐449a‐5p	Rheumatoid arthritis	T cells	Notch1 pathways	Arthritis clinical score + T cell subset balance + bone erosion: clinical score, paw thickness, Th17/Treg (FCM), IL‐17A/IL‐10 (ELISA), bone erosion (micro‐CT)	Modulate Notch1 signaling and restore cell balance, ameliorate the development and severity of arthritis	Chen et al. [40]
Mice, spleen, inguinal and axillary lymph nodes	dUC + sucrose/D2O cushion	TEM, NTA, western blot (CD9, CD63, CD81)	Treg‐Exos	50–150 nm	sEVs	miR‐709	SCI	Microglia	NKAP	Motor score + microglia pyroptosis: BMS score, footprint analysis, NLRP3/GSDMD/caspase‐1 (western blot), IBA1^+^/GSDMD^+^ (IF)	Inhibit microglia pyroptosis and improve motor recovery after SCI by targeting NKAP	Xiong et al. [11]
Mice, spleen, inguinal and axillary lymph nodes	dUC + sucrose/D2O cushion	TEM, NTA, western blot (CD9, CD63, CD81)	Treg‐Exos	40–150 nm	sEVs	miR‐2861	SCI	bEND.3 cells	IRAK1	BSCB integrity + motor score: occludin/ZO‐1 (IF/western blot), TEER/FITC‐dextran permeability, BMS score, MEP electrophysiology	Affect BSCB integrity and motor function after SCI by targeting IRAK1	Kong et al. [12]

*Note*: bEND.3 cells, a mouse brain microvascular endothelial cell line; dnIKK2, dominant negative variant of IKK2; YAMC cells, conditionally immortalized murine colonic epithelial cells.

Abbreviations: BSCB, blood–spinal cord barrier; CCND1, cyclin D1; CCNE1, cyclin E1; DCs, dendritic cells; DLS, dynamic light scatter; dUC, differential ultracentrifugation; ELISA, enzyme‐linked immunosorbent assay; EVs, extracellular vesicles; FCM, flow cytometry; IBD, inflammatory bowel disease; NKAP, NF‐κB activating protein; NTA, nanoparticle tracking analysis; SCI, spinal cord injury; sEVs, Small EVs; TEM, transmission electron microscope; Treg‐EVs, Treg‐derived EVs; ZO‐1, zonula occludens‐1.

EVs are known to be vital in enabling intercellular communication by transporting various substances, such as proteins, mRNAs, miRNAs, lipids, and small molecule metabolites from the parent cells [45, 46]. These EVs are recognized to play roles in various physiological and pathological conditions, such as autoimmune diseases and transplant rejection. In addition to their functional roles, EVs offer distinct advantages, including good stability, targeted properties, and favorable biocompatibility. These qualities have generated significant potential for use in different fields of medicine, including drug delivery, disease treatment, and disease diagnosis [47, 48].

Compared to other lymphocytes, Tregs have been observed to release more CD63^+^ exosomes (Treg‐Exos) following activation [9]. These exosomes have been demonstrated to exhibit immunosuppressive effects via several mechanisms. Furthermore, analysis has detected Tregs immunosuppressive proteins, including IL‐10, CTLA‐4, CD25, CD73, and other molecules involved in immune regulation [49, 50]. IL‐10 and CTLA‐4 are essential molecules involved in the immunosuppressive functionality of Tregs. However, studies have shown that CTLA‐4 levels are lower in Treg‐Exos, indicating nonenrichment meaning that CTLA‐4 is unlikely to account for most of the observed effects. CD73, found in Treg‐Exos, has been demonstrated to protect cells from immune damage by hydrolyzing exogenous 5′‐AMP to produce adenosine [50]. The adenosine generated can bind to the A2a receptor on Teffs. This process increases the levels of intracellular cAMP, inhibits its activity, reduces the production of cytokines such as IL‐2 and IFN‐γ, and helps to avoid excessive immune damage [51]. In addition, adenosine promotes the generation of adaptive Treg cells, which enhance immune tolerance by inhibiting IL‐6 and increasing TGF‐β [52]. Recent research has also shown that Foxp3^+^ Tregs secrete sEVs containing neuropilin‐1 (Nrp1), which can inhibit the proliferation of Teffs and regulate their gene expression in vitro in an Nrp1‐dependent manner; this process is related to the promotion of skin graft tolerance [53]. In another study, Sullivan et al. [54] identified a novel mechanism involving EV‐related IL‐35 in tolerance to infection. Tregs, iTr35, and B cells can release EVs that carry Ebi3 and p35, thus enabling them to target and infect a greater number of T and B lymphocytes. This process leads to the expression of IL‐35 on the surface of infected cells, thus promoting the exhaustion of non‐Treg cells and the secondary effects of immunosuppression.

## 3. miRNAs in Treg‐EVs

### 3.1. Biological Characteristics of miRNAs

miRNAs are small, evolutionarily conserved noncoding RNA molecules that regulate gene expression by binding to the 3′‐UTR of target mRNAs, and sometimes also to the 5′‐UTR of certain mRNAs. This interaction leads to either the degradation of mRNA or the suppression of translation, thereby playing vital roles in various cellular processes, including cell proliferation, differentiation, apoptosis, and development [55, 56]. Recent studies have shown that the expression levels of circulating miRNAs are linked to pathological status, and that some miRNAs may serve as potential biomarkers for disease diagnosis and prognosis [57]. In addition, alterations in miRNA expression can disrupt multiple biological processes by impacting the silencing of target genes, which are linked to the development and progression of various diseases, such as RA and MS, as well as transplant rejection. Therefore, therapies based on miRNA have significant potential for clinical use, including diagnosis and gene treatment [58]. However, the investigation and use of naked miRNA‐based agents have been significantly limited due to their short circulation time, poor targeting ability, and off‐target effects [59]. EVs can shield encapsulated miRNAs from degradation by ribonucleases (RNases) that are present in body fluids. Furthermore, because they are composed of self‐derived components, these vesicles exhibit low antigenicity and toxicity. Consequently, EVs are promising carriers for nucleic acid drugs, including miRNAs [60].

### 3.2. Immune Effects of miRNAs Derived From Treg‐EVs

**Figure 2:**
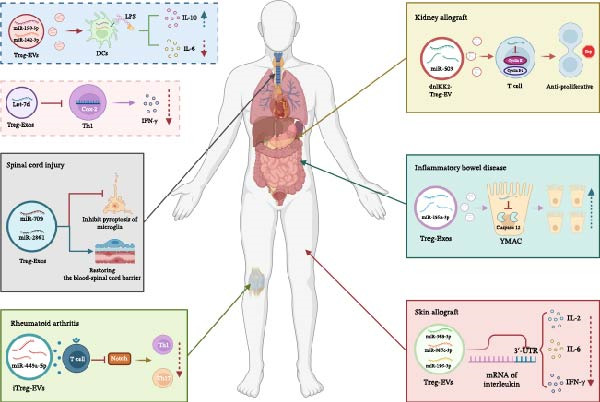
Schematic diagram of the application of Treg‐EVs‐derived miRNAs in immune‐related diseases. DCs, dendritic cells; LPS, lipopolysaccharide; miRNAs, microRNAs; Treg‐EVs, T cells‐derived extracellular vesicles; YAMC, conditionally immortalized murine colonic epithelial.

Recently, an increasing body of research has shown that Treg‐EVs can also support the immune response by carrying various miRNAs, including let‐7d, miR‐150‐5p, miR‐449a‐5p, miR‐709, and miR‐2861 (Table 2 and Figure 2). These miRNAs can influence immune cells and have the potential to provide therapeutic effects in managing various immune‐related diseases and recovery from injury.

Okoye et al. [9] demonstrated that Treg cells inhibit T cell‐mediated diseases through a non‐cell‐autonomous gene silencing mechanism involving exosomes containing miRNA. These authors revealed this process by using defective Treg cells that disrupted miRNA biogenesis or the exosomal pathway. Additionally, transcriptional analysis and miRNA inhibitor studies indicated that the presence of let‐7d in Treg‐Exos could suppress Th1 cell proliferation and IFN‐γ secretion by targeting the *Cox-2* gene, thereby reducing Th1 cell‐mediated inflammation. In addition to T cells, DCs have also been found to act as target cells for the miRNAs in Treg‐EVs. Research by Tung et al. [10] showed that miRNAs can be transferred from Tregs to DCs via Treg‐EVs, with a particular focus on two specific miRNAs, miR‐150‐5p, and miR‐142‐3p, which are crucial to this transfer process. Once DCs ingest miR‐150‐5p and miR‐142‐3p carried by Treg‐EVs, they respond to lipopolysaccharide (LPS) stimulation with increased IL‐10 production and a simultaneous reduction in IL‐6 production. The results of these studies suggest that Treg‐EVs may help to induce a tolerogenic phenotype in DCs by transferring miRNAs.

SCI usually occurs after trauma and often leads to a significant loss of functionality in the limbs below the affected area; in some cases, SCI can also be fatal [61, 62]. Following SCI, both innate and adaptive immune cells are activated and play crucial roles in the clearance of debris and the reduction of inflammation [63]. The presence of miR‐709 and miR‐2861 in Treg‐Exos can reduce microglial pyroptosis and promote the repair of the BSCB, respectively, thus facilitating the recovery of motor function [11, 12]. A previous study investigating the link between Tregs and pyroptosis in microglia used bioinformatic analyses to demonstrate a significant enrichment of miR‐709 in Treg cells and Treg‐derived exosomes, with NF‐κB activating protein (NKAP) identified as a target gene for miR‐709. Further experiments revealed that Treg cells used exosomal miR‐709 to target NKAP, thereby reducing microglial pyroptosis and facilitating the recovery of motor function after SCI. In addition, modified exosomes overexpressing miR‐709 were shown to reduce inflammation and improve motor recovery after SCI [11]. Another study focusing on Tregs and the restoration of the BSCB after SCI reported that Treg‐Exos could transfer miR‐2861 to the target and suppress the IL‐1 receptor‐associated kinase 1 (IRAK1) gene. This regulatory ability subsequently influenced the expression of vascular tight junction proteins, thereby impacting the integrity of the BSCB and motor functionality in a mouse model of SCI [12].

## 4. Treg‐EVs and Their miRNAs in Immune‐Related Diseases

### 4.1. Autoimmune Diseases

#### 4.1.1. RA

RA is a common chronic autoimmune disease characterized by symmetrical joint lesions, often leading to joint damage and deformity [64, 65]. The pathogenesis of RA is complex, and the disrupted autoimmune tolerance caused by the dysfunctionality of Treg cells may be closely linked to the onset and progression of the disease [66, 67]. Therefore, increasing the number of Treg cells and regaining their functionality is considered as a very effective treatment strategy [68]. A previous study involving a rat model of collagen‐induced arthritis (CIA) demonstrated that dihydroartemisinin (DHA) treatment promoted the differentiation of IL‐10^+^ and TGF‐β1^+^ Tregs, thereby strengthening its inhibitory effect on osteoclast differentiation [69]. Moreover, DHA was found to reduce aggressive bone degeneration by restoring Treg cells in the spleen and joint‐draining lymph nodes. In addition, proteomic analysis revealed that DHA induced changes in the proteome of Treg cells in the popliteal lymph nodes, helping to maintain immune tolerance at local joints and further slow the progression of RA by generating autologous Treg cells that expressed alloantigen Col2a1 (type II collagen alpha 1 chain) and CD8a (T‐cell surface glycoprotein CD8 alpha chain). In another study, Chen et al. [40] revealed the potential of Treg‐EVs for the treatment of RA. iTreg‐EVs induced by TGF‐β were shown to selectively target the affected joints and inhibit the expression of Notch1 in recipient T cells by transferring miR‐449a‐5p. Notch signaling has been shown to exert significant effect on the differentiation and functionality of T cells. The Notch pathway has been shown to inhibit the development of Th1 and Th17 cell populations, with only minimal impact on the development of Treg cells [70]. Therefore, restoration of the balance between Th17 and Treg cells in a mouse model of CIA via iTreg‐EVs was considered to be due to the inhibition of Notch1 by miR‐449a‐5p, which slowed the progression of RA and relieved its symptoms [40]. Consequently, iTreg‐EVs and the miRNAs they contain, provide a potential non‐Treg cell‐dependent approach for the treatment of RA.

#### 4.1.2. IBD

IBD is a chronic and recurring inflammatory condition of the intestines, with two sub‐types: Crohn’s disease (CD) and ulcerative colitis (UC) [71]. CD can lead to transmural inflammation, often accompanied by complications such as intestinal granuloma, fistula, and stenosis. UC typically presents as mucosal inflammation limited to the rectum and colon [72, 73]. The exact cause of IBD remains unclear and may be associated with genetic predisposition, environmental factors, intestinal microbiota, and immune reactions [74]. It has been shown that the proportion of Treg cells exhibiting the CD4^+^CD25^+^Foxp3^+^ phenotype is reduced in IBD patients compared to healthy controls, and that the proportion of Treg cells in patients with active IBD is also lower compared to patients in remission [75]. Wang et al. [76] suggested that the adoptive transfer of Tregs could stabilize the expression of Foxp3 through the Smad3 and NFAT2 pathways, which in turn influenced Th17/Treg differentiation and alleviated colitis in a mouse model. Do et al. [77] discovered that the activation of IL‐27 could enhance the expression of Lag3 on Tregs. Lag3 has the potential to limit the maturation of Tregs via interactions with MHC II molecules on DCs, which in turn inhibits the activation of developing T cells and diminishes the intestinal inflammatory response. Therefore, targeting the IL‐27/Lag3 axis mediated by Tregs could offer new approaches for the treatment of IBD. Liao et al. [39] discovered that Treg‐Exos could be transferred to conditionally immortalized murine colonic epithelial cells (YAMC) and inhibit the expression of Caspase 12 via miR‐195a‐3p in Treg‐Exos, thus promoting proliferation and reducing apoptosis in YAMC cells. In experimental models, the administration of Treg‐Exos has been observed to increase the expression of the tight junction protein zonula occludens‐1 (ZO‐1) in mouse colon tissue, while simultaneously reducing the levels of inflammatory mediators TNF‐α, IL‐1β, and IL‐6. This process helped to restore the structural integrity of the colon, reduce mucosal damage, and consequently improve dextran sodium sulfate‐induced IBD in a mouse model [39]. Based on the findings from these animal studies, future research may focus on developing Treg‐Exos delivery systems loaded with specific miRNAs and further validating their efficacy and safety in preclinical and clinical trials.

#### 4.1.3. Autoimmune Vasculitis

Autoimmune vasculitis is an inflammatory condition that affects the walls of blood vessels, leading to tissue damage. This type of inflammation can cause either destruction or alteration of the vascular walls, which can then impact the lumen of the blood vessels. The pathological changes in these vessels subsequently hinder the delivery of essential nutrients and oxygen to nearby tissues, potentially resulting in organ damage and cell death [78, 79]. The varying numbers and functions of Treg cells are closely associated with the progression of different types of vasculitides. For example, certain Tregs subsets, such as CD8^+^ Tregs, have been found to be defective in giant cell arteritis (GCA). Wen et al. [80] demonstrated that CD8^+^ Tregs can produce exosomes containing NADPH oxidase 2 (NOX2). These exosomes subsequently fuse with CD4^+^ T cells, thereby disrupting CD4^+^ T cell signaling by producing reactive oxygen species to subsequently inhibit their activation and proliferation. In patients with GCA, abnormalities in Notch4 signaling were observed in CD8^+^ Treg cells; these abnormalities caused a change in the subcellular localization of NOX2 in these Treg cells. NOX2 was more frequently directed into early and circulating endosomes rather than being release directly into the exosome. Consequently, the ability of CD8^+^ Treg cells to suppress the activation and expansion of CD4^+^ T cells was reduced due to the transfer of NOX2 through exosomes [81]. Furthermore, vascular endothelial growth factor, which is abundant in the blood of patients with GCA, has been shown to induce endothelial cells to express Jagged1, a ligand of Notch. This, in turn, activated CD4^+^ T cells expressing the Notch1 receptor and promoted their differentiation into Th1 and Th17 cells, which are tissue‐invasive and can induce inflammation in blood vessels [82]. Therefore, inhibiting NOTCH4 signaling may restore the suppressive function of CD8^+^ Tregs by transferring NOX2 to CD4^+^ T cells via exosomes, thereby protecting arteries from inflammation [81].

#### 4.1.4. MS

MS is a chronic inflammatory disease linked to autoimmunity that can cause demyelination and neurodegeneration of the central nervous system [83]. MS is more common in females than males, with a larger gender difference seen during adolescence [84, 85]. The etiology and pathogenesis of MS are complex and may be linked to genetic and environmental factors [86]. A previous study reported a reduction in both the number and function of Tregs in the peripheral blood of patients with MS; subsequent investigations also revealed that the expression profile of miRNAs in circulating exosome samples taken from the blood of MS patients exhibited significant differences compared to a control group [87]. Notably, high expression levels of let‐7i were detected; let‐7i can suppress the induction of Tregs by targeting the insulin‐like growth factor 1 receptor and the transforming growth factor beta receptor 1, thereby contributing to the development of MS [87]. Furthermore, defects in the inhibitory effects of Treg‐Exos have been linked to the pathogenesis of MS. Azimi et al. [15] isolated and cultured Tregs from both MS patients and healthy controls and then purified Treg‐Exos from culture media. After co‐culturing with Teffs, the authors found that Treg‐Exos from MS patients exhibited a diminished ability to inhibit the proliferation of Teffs and a lower tendency to induce their apoptosis compared to the control group. The underlying causes of Treg‐Exos dysfunctionality in MS patients have yet to be identified, and potential therapeutic interventions aimed at restoring the inhibitory function of Treg‐Exos also needs to be investigated.

### 4.2. Transplantation

Acute and chronic immune rejection continue to be the main obstacles to long‐term graft survival in organ transplantation [88–90]. However, the strong immunosuppressive effects of Tregs have sparked significant research interest in Treg‐EVs and their miRNA cargo, highlighting these biological components as a promising therapeutic approach to address transplant rejection.

#### 4.2.1. Kidney Allografts

Immune rejection remains as a major threat to the survival of kidney allografts despite immunosuppressive treatments, spurring research into new strategies such as exosome‐based immunomodulation to improve tolerance and reduce treatment‐related toxicity [91–93]. Using a rat model, Yu et al. [94] performed serum analysis and histological investigations to demonstrate that treatment with Treg‐derived exosomes via the tail vein delayed acute allograft rejection, and that this intervention significantly contributed to prolonging the survival of kidney allografts. Furthermore, the mixed lymphocyte reactions in vivo indicated that Treg‐derived exosomes could suppress T cell proliferation in a dose‐dependent manner and was likely involved in Treg cell‐mediated immune tolerance. The results of this study suggested that exosomes released from Treg cells, especially those from donor‐type Tregs, might serve as potential immunosuppressive agents for the treatment of transplant rejection. However, the precise mechanism of immune tolerance in this process has yet to be elucidated. Another study demonstrated that EVs obtained from dominant negative variant of IKK2 (dnIKK2)‐Tregs are linked to a specific subset of CD4^+^ CD25^−^ Tregs generated in vitro. This occurred when rat allogeneic DCs were rendered immature by inhibiting NF‐κB through the adenoviral gene delivery of the dnIKK2, which also extended the survival of transplanted kidneys by reducing T cell proliferation [37]. EVs released by dnIKK2‐Tregs contain specific miRNAs and inducible nitric oxide synthase (iNOS), especially miR‐503, along with iNOS mRNA and its protein. These factors impact T cells by disrupting cell cycle progression, promoting apoptosis, and converting target T cells into Tregs. Notably, miR‐503 was shown to influence cell cycle regulation in T cells by reducing the levels of Cyclin E and Cyclin D1, leading to T cell cycle arrest [37]. Based on these findings from animal studies, future research should focus on further validating these effects and translating results into clinical applications.

#### 4.2.2. Skin Allografts

Similar to the situation seen in kidney allografts, current research on skin allografts also aims to develop effective immune tolerance strategies to enhance graft survival and reduce the risk of rejection [95]. A recent investigation into the impact of sEV secreted by Tregs has revealed that wild‐type (wt) Treg‐derived sEVs (wt sEV) can suppress the proliferation of Teffs and influence their phenotypes (such as the induction of Foxp3 expression) in a Nrpl‐dependent manner in vitro [53]. In vivo, the local injection of wt sEV significantly extended the survival of mouse skin allografts and reduced the ratio of M1 to M2 macrophages in grafts. In contrast, *Nrpl* knockout Treg cell‐derived sEV had no such effect [53]. Tung et al. [38] specifically investigated how sEV is released by human Tregs in immune regulation and skin allografts. In a humanized mouse model of human skin transplantation, Treg‐EVs significantly reduced immune cell infiltration and reduced skin allograft damage, and by isolating and activating human Treg cells, sEVs suppressed the proliferation of Teffs in a dose‐dependent manner, and modified cytokine production, thus reducing the levels of pro‐inflammatory cytokines IL‐2, IL‐6, and IFN‐γ, while increasing the levels of anti‐inflammatory cytokines IL‐4 and IL‐10 [38]. Notably, miRNA analysis revealed that Treg‐EVs were enriched with miRNAs unique to Treg‐EVs, including miR‐369‐3p, miR‐376c‐3p, and miR‐195‐3p. Bioinformatic analysis further demonstrated that miRNAs in the Treg‐EVs indirectly inhibited the expression of pro‐inflammatory factors by targeting the 3′‐UTR region of the mRNAs for IL‐6, IL‐2, and IFN‐γ [38]. Therefore, by miRNA‐mediated cytokine regulation, Treg‐EVs may serve as a novel cell‐free therapy for inhibiting graft rejection.

## 5. Integrative Analysis: Shared Key Mechanisms and Dual Roles

### 5.1. Shared Key Regulatory Pathways

**Figure 3:**
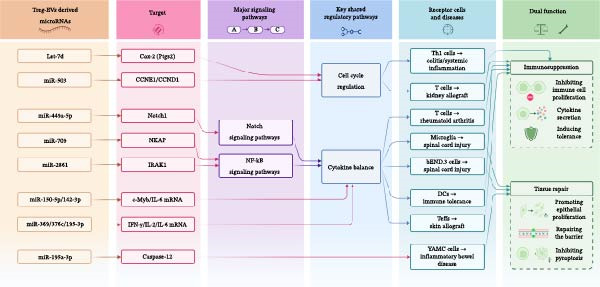
A graphical summary of the immunomodulatory profile of Treg‐EVs‐derived miRNAs in immune‐related diseases. This diagram summarizes the key miRNAs derived from Treg‐EVs, their molecular targets, major signaling pathways, and recipient cells involved in various immune‐related diseases and highlights their dual role in immunosuppression through regulating cell cycle and cytokine balance. bEND.3 cells, a mouse brain microvascular endothelial cell line; CCND1, cyclin D1; CCNE1, cyclin E1; DCs, dendritic cells; YAMC cells, conditionally immortalized murine colonic epithelial cells.

Various immune‐related diseases have been discussed in the preceding sections of this review. It is important to note that these mechanisms appear to share several common regulatory mechanisms, particularly in the contexts of cell cycle regulation and cytokine balance (Figure 3). First, Treg‐EVs can regulate the growth of recipient cells by binding to specific genes to influence cell proliferation or tissue repair. For example, let‐7d inhibits the proliferation of Th1 cells by inhibiting Cox‐2, while miR‐503 induces cell cycle arrest in T cells by downregulating the CCNE1 and CCND1 cyclins, both of which can achieve the fundamental inhibition of effector cell expansion [9, 37]. Second, Tregs can regulate the functionality of immune cells by targeting related genes, affecting signaling pathways, or by changing the expression of specific inflammatory gene factors. Specifically, miR‐449a‐5p, by targeting the Notch1 pathway, and miR‐709 and miR‐2861, by inhibiting the NF‐κB regulatory proteins NKAP and IRAK1, respectively, have been shown to rewire the inflammatory response program of T cells or microglia from the signal transduction node [11, 12, 40]. In addition, miRNAs, such as miR‐150‐5p, miR‐142‐3p and miR‐369‐3p, precisely reduce the production of inflammatory mediators at the post‐transcriptional level by directly binding to the mRNAs of cytokines such as c‐Myb and IL‐6 [10]. In tissue repair scenario, miR‐195a‐3p has been shown to enhance the survival and proliferation of colonic epithelial cells by inhibiting Caspase 12 [39]. This multilevel and synergistic mode of action is the core mechanism by which Treg‐EVs can restore immune homeostasis across diseases: from the inhibition of proliferation and the reprogramming of signals to the direct silencing of effector molecules, and then the promotion of tissue repair.

### 5.2. Dual Roles: Immunosuppression Versus Tissue Repair

The mechanistic work described above also suggests that the functionality of Treg‐EVs exerts significant plasticity. In addition to the differences in miRNAs carried by Treg‐EVs, which are crucial for their functional performance, their final effects are also highly dependent on the type of recipient cells and the disease microenvironment. When Treg‐EVs deliver their miRNA cargo to immune cells (such as T cells and DCs), they predominantly play a classical immunosuppressive role, which is manifested by the inhibition of cell activation, proliferation, and the production of proinflammatory cytokines, which continues the core functionality of Treg cells to maintain peripheral tolerance. However, when the same or different Treg‐EV populations act on non‐immune stromal cells or barrier cells, their functions shift to active tissue repair and reconstruction (Figure 3). For example, in IBD, miR‐195a‐3p is delivered to colonic epithelial cells to promote mucosal barrier repair through anti‐apoptotic effects [39]. Following SCI, miR‐2861 is delivered to microvascular endothelial cells in the brain to repair the BSCB by enhancing the expression of tight junction proteins [12]. This dual role highlights the therapeutic potential of Treg‐EVs not only in autoimmune diseases, but potentially also in injury and regenerative medicine.

## 6. Conclusion and Future Prospects

In this review, we summarized recent studies relating to the potential and feasibility of Treg‐EVs and their miRNAs to facilitate the treatment of immune‐related diseases. As conserved mediators of intercellular communication, miRNAs are increasingly viewed as key regulators of immune homeostasis and promising therapeutic targets. EVs, which have low immunogenicity and can protect encapsulated miRNAs from enzymatic degradation, have become excellent delivery vehicles for therapies involving nucleic acids. Treg‐EVs can support the immunosuppressive functionality of Treg cells and exhibit significant therapeutic potential through their miRNAs. Beyond a descriptive summary, we have integrated findings into a mechanistic network that reveals shared pathways (e.g., Notch, NF‐κB, and the cell cycle) and dual roles (immunosuppression vs. tissue repair). This integrative framework highlights how seemingly disparate miRNAs converge on common biological processes to restore immune homeostasis.

Nevertheless, current research in this area still faces many challenges. First, as mentioned earlier, the isolation and purification techniques used for EVs have yet to be standardized. The differential biogenesis of Treg‐EVs subtypes implies functional differences, and even though a preliminary subtype classification is recommended according to the MISEV2023 guidelines, determining their precise functional differences remains challenging. Second, in related research, Treg‐EVs from various sources and processed by different methods, such as ultracentrifugation, SEC, and filtration, show significant heterogeneity, a critical translation gap reflecting the lot‐to‐lot variability of Tregs sources that complicates reproducibility. For instance, the expression profiles of Treg‐EVs miRNAs differ significantly between humans and mice. Even within the same species, their profiles can vary after different pretreatments. These issues make it difficult to directly compare and summarize results across different experiments, which may affect the overall applicability and representativeness of the findings.

In addition, further analysis is needed on the mechanisms underlying miRNA‐targeted delivery, particularly regarding the quantitative burden of miRNAs per Treg‐EVs and stoichiometric sufficiency at recipient cells to elicit robust target silencing. Rigorous validation is essential to distinguish EV‐mediated miRNA transfer from the confounding effects of co‐isolated cytokines, LPS, lipoproteins, or protein aggregates. This requires demonstration of RNase/protease protection, membrane disruption controls, careful interpretation of chemical inhibition studies (e.g., GW4869 off‐target effects), and spike‐in controls with dose‐response relationships. The transcellular network and miRNA changes in pathological environments also warrant investigation, such as whether the functional deficiencies of Treg‐EVs in MS patients correlate with alterations in specific miRNA expression profiles.

Finally, bridging from bench to bedside requires us to address critical translation gaps, including establishing optimal dosing regimens, determining administration routes, and developing GMP/scale‐up manufacturing processes. Overcoming technical barriers, such as biodistribution variability, macrophage uptake, endosomal escape, dose, scalability, and safety, also remain as significant challenges.

These disparities and challenges suggest that the functions of Treg‐EVs‐miRNAs cannot be simply extrapolated to different diseases. Rather, we need to specifically evaluate the origin, properties, cargo content, dosage, responsiveness of receptor cells, and the impact characteristics of the pathological microenvironment associated with Treg‐EVs. Future research endeavors should be guided by this holistic viewpoint to devise joint targeting strategies for pivotal network hubs.

Collectively, Treg‐EVs and their miRNAs show significant potential in treating immune‐related diseases; however, further research is needed before they can be applied clinically.

## Author Contributions

Yudong Gao and Yuwen Mao conceived the study concept and design. Fengbo Zhang provided essential resources, supervised the study, oversaw project administration, and secured the funding that supported this work. Juncheng Zhou was responsible for data curation and visualization. Yudong Gao and Fengbo Zhang drafted the original manuscript. All authors—including Yudong Gao, Juncheng Zhou, Yuwen Mao, Mingxuan Yuan, Reyihanguli Yimin, and Fengbo Zhang—contributed to the critical revision and editing of the manuscript.

## Funding

This work was supported by the State Key Laboratory of Pathogenesis, Prevention and Treatment of High Incidence Diseases in Central Asia Fund (Grant SKL‐HIDCA‐2022‐JZ6), the Henan Key Laboratory of Immunology and Targeted Drugs Project (Grant HNKLITD2022001), and the National College Students Innovation and Entrepreneurship Training Program (Grant 202410760007).

## Disclosure

All the authors have read and approved the final manuscript and agree to be accountable for all aspects of the research.

## Ethics Statement

No ethical approval was required for this study.

## Conflicts of Interest

The authors declare no conflicts of interest.

## Data Availability

Data sharing is not applicable to this article as no datasets were generated or analyzed during the current study.
